# Fracture of a Rib with Aggravation and Relief from Hypericum

**Published:** 1889-05

**Authors:** B. L. B. Baylies


### FRACTURE OF A RIB, WITH IMMEDIATE AGGRAVATION AND SPEEDY RELIEF FROM HYPERICUM$^{900}$.

A boy eleven years of age, while playing in the street was run down by a coupé, which was seen to pass over the lower portion of his chest. A neighboring physician temporarily prescribed for him. I saw him two hours after the accident, and found him prostrate, but quite conscious and intelligent; skin cool, much pallor of the face; pulse rather slow; respiration 46, inspirations partially repressed, painful and grunting; expectoration of blood; pains acute and pricking in the region of the lower ribs of each side, especially of the eighth rib, right side, which was very sensitive to pressure at about two and one-half inches from its cartilage. After applying the bandage to the chest, Aconite$^{200}$ and afterward Arnica $^{200}$ were given without relief. The shock and the pricking character of the pains, and probable puncture of the lung suggested Hypericum perfol., a solution of the 900th (Fincke) of which was given, and was only in the mouth when the patient quickly drew up his limbs, exclaimed oh! oh! and suffered much aggravated pain in the injured parts; this gradually subsided, and he slept at intervals; but pain returning, took another dose an hour and a half after the first, followed immediately by similar sharp aggravation, but subsiding like the former. In two hours from the first dose of Hypericum, respiration had declined from 46 to 33; he passed fairly well the latter half of the night, sleeping at one time an hour, having been turned, at his request, partially on the injured side. The following day, bloody expectoration continuing, he took, in solution, Millefolium$^{200}$, which also has stitches in the "lower right ribs," "in the left false ribs," etc.; this seemed to arrest expectoration of blood. On the tenth day after injury, Symphytum was given. Fifteenth day, he feels quite well. Some callous swelling of the injured portion of the rib. Recovered without further trouble. The immediate aggravation from Hypericum$^{900}$, and the rapid reaction, with the relief following it, were remarkable.

B. L. B. Baylies.

